# Supplementary material for: A Novel Widespread MITE Element in the Repeat-Rich Genome of the Cardinium Endosymbiont of the Spider Oedothorax gibbosus
Source: Microbiol Spectr. 2022 Oct 27;10(6):e02627-22. doi: 10.1128/spectrum.02627-22 (PMC9769881; doi:10.1128/spectrum.02627-22)
Supplement: Supplemental file 2 — Fig. S1. Download spectrum.02627-22-s0002.pdf, PDF file, 0.2 MB [file spectrum.02627-22-s0002.pdf]

```
ISCca4               ACCTAAGTTCGGGATTAGCTTGTTAAAAAATAGCTTTAAAAAGCTCCTTTGTGTTTTAAATTTGGAGATAAAAAAATACTAACCAGATTATCCAAAGGAGCCAGTTATGAATGTAGAAAAATTAGTTGAAATATATTATGCTGTTGATGA  150
ISCca4_CDS           ----------------------------------------------------------------------------------------------------------ATGAATGTAGAAAAATTAGTTGAAATATATTATGCTGTTGATGA   44
ISCca4_IRL           ACCTAAGTTCGGGATTAGC-----------------------------------------------------------------------------------------------------------------------------------   19
ISCca4_IRR           ------------------------------------------------------------------------------------------------------------------------------------------------------
MITECca01            ACGTGAATTCGGGATTAGATTATT------------------------------------------------------------------------------------------------------------------------------   24
MITECca01_IRL        ACGTGAATTCGGGATT--------------------------------------------------------------------------------------------------------------------------------------   16
MITECca01_IRR        ------------------------------------------------------------------------------------------------------------------------------------------------------
MITECca02            ACCTGAGTTCGGGATTAGCTTGTTAAAAAATAGCTTTAAAAAGCTTCTTTGTGTTTTAAATTTGGAGATAAAAAAATACTAACCAGATTATCCAAAGGAGCCAGT---------------------------------------------  105
MITECca02_IRL        ACCTGAGTTCGGGATT--------------------------------------------------------------------------------------------------------------------------------------   16
MITECca02_IRR        ------------------------------------------------------------------------------------------------------------------------------------------------------
ISCca4_MITE-like     ACCTAAGTTCGGGATTAGCTTGTTAAAAAATAGCTTTAAAAAGCTTCTTTGTGTTTTAAATTTGGAGATAAAAAAATACTAACCAGATTATCC---------------------------------------------------------   93
ISCca4_MITE-like_IRL ACCTAAGTTCGGGATT--------------------------------------------------------------------------------------------------------------------------------------   16
ISCca4_MITE-like_IRR ------------------------------------------------------------------------------------------------------------------------------------------------------
                     1.......10........20........30........40........50........60........70........80........90.......100.......110.......120.......130.......140.......150
```

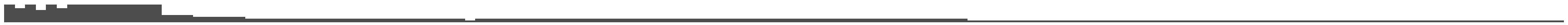

```
ISCca4               ATTTCTCATAAAGTTTATGCCGTATATGGAAAAACAACTGTTAACCAACTCTAAAAGAAAACCCACTAGAACTTGTTCATTAACTTTGAGTGAAATAATGACTGTTCTTATTGCCTTTCATGTGATTGGTTTTAGAAATTTTAAGTCTTA  300
ISCca4_CDS           ATTTCTCATAAAGTTTATGCCGTATATGGAAAAACAACTGTTAACCAACTCTAAAAGAAAACCCACTAGAACTTGTTCATTAACTTTGAGTGAAATAATGACTGTTCTTATTGCCTTTCATGTGATTGGTTTTAGAAATTTTAAGTCTTA  194
ISCca4_IRL           ------------------------------------------------------------------------------------------------------------------------------------------------------   19
ISCca4_IRR           ------------------------------------------------------------------------------------------------------------------------------------------------------
MITECca01            ------------------------------------------------------------------------------------------------------------------------------------------------------   24
MITECca01_IRL        ------------------------------------------------------------------------------------------------------------------------------------------------------   16
MITECca01_IRR        ------------------------------------------------------------------------------------------------------------------------------------------------------
MITECca02            ------------------------------------------------------------------------------------------------------------------------------------------------------  105
MITECca02_IRL        ------------------------------------------------------------------------------------------------------------------------------------------------------   16
MITECca02_IRR        ------------------------------------------------------------------------------------------------------------------------------------------------------
ISCca4_MITE-like     ------------------------------------------------------------------------------------------------------------------------------------------------------   93
ISCca4_MITE-like_IRL ------------------------------------------------------------------------------------------------------------------------------------------------------   16
ISCca4_MITE-like_IRR ------------------------------------------------------------------------------------------------------------------------------------------------------
                     .......160.......170.......180.......190.......200.......210.......220.......230.......240.......250.......260.......270.......280.......290.......300
```

```
ISCca4               TTATATTCATTTACAGCAATTTCATTCTAGTAAATTTGGAAAGTTAGTAAGATACAATAGATTTATAGAACTGATCCAAAGAACGTTAGTTCCCTTGTACTGTTTTACACAAAGTCTTTCTAAAACAAAAACAGGTTGTTATTTTATGGA  450
ISCca4_CDS           TTATATTCATTTACAGCAATTTCATTCTAGTAAATTTGGAAAGTTAGTAAGATACAATAGATTTATAGAACTGATCCAAAGAACGTTAGTTCCCTTGTACTGTTTTACACAAAGTCTTTCTAAAACAAAAACAGGTTGTTATTTTATGGA  344
ISCca4_IRL           ------------------------------------------------------------------------------------------------------------------------------------------------------   19
ISCca4_IRR           ------------------------------------------------------------------------------------------------------------------------------------------------------
MITECca01            ------------------------------------------------------------------------------------------------------------------------------------------------------   24
MITECca01_IRL        ------------------------------------------------------------------------------------------------------------------------------------------------------   16
MITECca01_IRR        ------------------------------------------------------------------------------------------------------------------------------------------------------
MITECca02            ------------------------------------------------------------------------------------------------------------------------------------------------------  105
MITECca02_IRL        ------------------------------------------------------------------------------------------------------------------------------------------------------   16
MITECca02_IRR        ------------------------------------------------------------------------------------------------------------------------------------------------------
ISCca4_MITE-like     ------------------------------------------------------------------------------------------------------------------------------------------------------   93
ISCca4_MITE-like_IRL ------------------------------------------------------------------------------------------------------------------------------------------------------   16
ISCca4_MITE-like_IRR ------------------------------------------------------------------------------------------------------------------------------------------------------
                     .......310.......320.......330.......340.......350.......360.......370.......380.......390.......400.......410.......420.......430.......440.......450
```

```
ISCca4               TGCAACAGCCATCAAGGTTTGCCATATCAAAAGAGCCTATACGCACAGGGTTTTTAAATCGATTGCTACTAAAGGTAAAACCAGCATCGGTTGGTTTTTTGGATTAAAGTTGCACTTGATAGTCAATGATTTAGGAGAAATTATGAATTT   600
ISCca4_CDS           TGCAACAGCCATCAAGGTTTGCCATATCAAAAGAGCCTATACGCACAGGGTTTTTAAATCGATTGCTACTAAAGGTAAAACCAGCATCGGTTGGTTTTTTGGATTAAAGTTGCACTTGATAGTCAATGATTTAGGAGAAATTATGAATTT   494
ISCca4_IRL           ------------------------------------------------------------------------------------------------------------------------------------------------------   19
ISCca4_IRR           ------------------------------------------------------------------------------------------------------------------------------------------------------
MITECca01            ------------------------------------------------------------------------------------------------------------TTTTTATTTGATAATCAAT-----------------------   43
MITECca01_IRL        ------------------------------------------------------------------------------------------------------------------------------------------------------   16
MITECca01_IRR        ------------------------------------------------------------------------------------------------------------------------------------------------------
MITECca02            ------------------------------------------------------------------------------------------------------------------------------------------------------   105
MITECca02_IRL        ------------------------------------------------------------------------------------------------------------------------------------------------------   16
MITECca02_IRR        ------------------------------------------------------------------------------------------------------------------------------------------------------
ISCca4_MITE-like     ------------------------------------------------------------------------------------------------------------------------------------------------------   93
ISCca4_MITE-like_IRL ------------------------------------------------------------------------------------------------------------------------------------------------------   16
ISCca4_MITE-like_IRR ------------------------------------------------------------------------------------------------------------------------------------------------------
                     .......460.......470.......480.......490.......500.......510.......520.......530.......540.......550.......560.......570.......580.......590.......600
```

```
ISCca4               TCAACTGACCACAGGTAAAACTAATGATAGGCTTCCTGTAGAAAATTTATGTAAACACTTCATGGGTAAAATGTTTGCCGATAAAGGTTACATAAGCAAAGATTTATTTGAAAAACTGATCGAGAAAGGCGTGGAACTCATTACCCAGAT   750
ISCca4_CDS           TCAACTGACCACAGGTAAAACTAATGATAGGCTTCCTGTAGAAAATTTATGTAAACACTTCATGGGTAAAATGTTTGCCGATAAAGGTTACATAAGCAAAGATTTATTTGAAAAACTGATCGAGAAAGGCGTGGAACTCATTACCCAGAT   644
ISCca4_IRL           ------------------------------------------------------------------------------------------------------------------------------------------------------   19
ISCca4_IRR           ------------------------------------------------------------------------------------------------------------------------------------------------------
MITECca01            -------------------------------------------------------------------------------------------ATTAATAAAAATTTAT-------------------------------------------   59
MITECca01_IRL        ------------------------------------------------------------------------------------------------------------------------------------------------------   16
MITECca01_IRR        ------------------------------------------------------------------------------------------------------------------------------------------------------
MITECca02            ------------------------------------------------------------------------------------------------------------------------------------------------------   105
MITECca02_IRL        ------------------------------------------------------------------------------------------------------------------------------------------------------   16
MITECca02_IRR        ------------------------------------------------------------------------------------------------------------------------------------------------------
ISCca4_MITE-like     ------------------------------------------------------------------------------------------------------------------------------------------------------   93
ISCca4_MITE-like_IRL ------------------------------------------------------------------------------------------------------------------------------------------------------   16
ISCca4_MITE-like_IRR ------------------------------------------------------------------------------------------------------------------------------------------------------
                     .......610.......620.......630.......640.......650.......660.......670.......680.......690.......700.......710.......720.......730.......740.......750
```

```
ISCca4               CAGAAAAAATATGAAAAATGCTTTTATGCCACTCTGGGATAAACTGATGCTTAGAAAACGATCTATAATTGAAACTATTATAGATCAGCTCAAAAATATTAGCCAGATAGAACATTCCAGGCATAGAAGCATACCTAATTTTCTAGTCAA   900
ISCca4_CDS           CAGAAAAAATATGAAAAATGCTTTTATGCCACTCTGGGATAAACTGATGCTTAGAAAACGATCTATAATTGAAACTATTATAGATCAGCTCAAAAATATTAGCCAGATAGAACATTCCAGGCATAGAAGCATACCTAATTTTCTAGTCAA   794
ISCca4_IRL           ------------------------------------------------------------------------------------------------------------------------------------------------------   19
ISCca4_IRR           ------------------------------------------------------------------------------------------------------------------------------------------------------
MITECca01            ----------------------------------------------------------------------------------------------AAAATTTGTTTTATAGACAGTTAAATAAGTAGTTGAATAACAAATAATTCTGATAA   115
MITECca01_IRL        ------------------------------------------------------------------------------------------------------------------------------------------------------   16
MITECca01_IRR        ------------------------------------------------------------------------------------------------------------------------------------------------------
MITECca02            ------------------------------------------------------------------------------------------------------------------------------------AACAAATTTT--------   115
MITECca02_IRL        ------------------------------------------------------------------------------------------------------------------------------------------------------   16
MITECca02_IRR        ------------------------------------------------------------------------------------------------------------------------------------------------------
ISCca4_MITE-like     -----------------------------------------------------------------------------------------------ATATTAGCCAGATAGAACATTCCAGGCATAGAAGCATACCTAATTTTCCAGTCAA   148
ISCca4_MITE-like_IRL ------------------------------------------------------------------------------------------------------------------------------------------------------   16
ISCca4_MITE-like_IRR ------------------------------------------------------------------------------------------------------------------------------------------------------
                     .......760.......770.......780.......790.......800.......810.......820.......830.......840.......850.......860.......870.......880.......890.......900
```

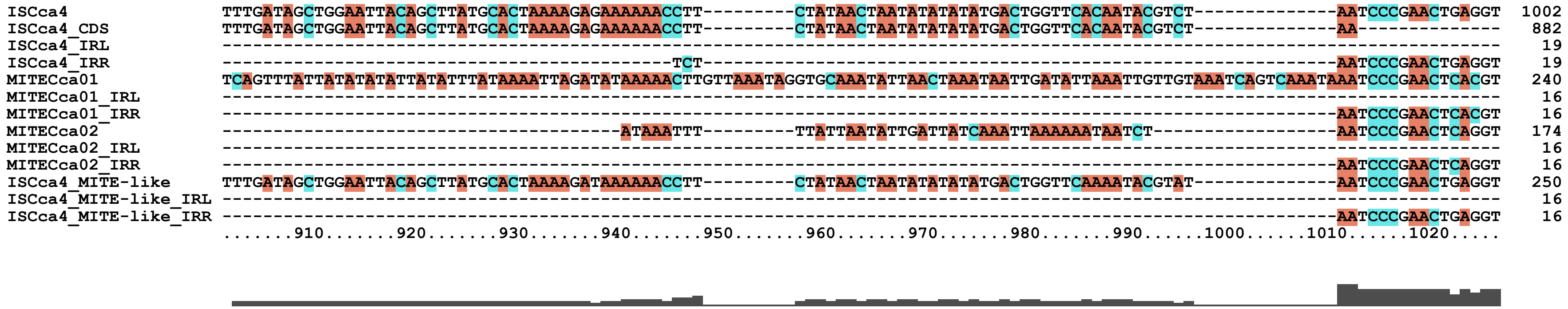

Figure S1. Alignment of ISCca4, MITE, and MITE-like element. Interleaved CLUSTAL-formatted alignment of ISCca4 and related mobile elements and their inverted repeats.
